# Integrated Colorimetric CRISPR/Cas12a Detection of Double-Stranded DNA on Microfluidic Paper-Based Analytical Devices

**DOI:** 10.3390/bios16010032

**Published:** 2026-01-01

**Authors:** Zhiheng Zhang, Qiyu Fu, Tiantai Wen, Youmin Zheng, Yincong Ma, Shixian Liu, Guozhen Liu

**Affiliations:** Integrated Devices and Intelligent Diagnosis (ID^2^) Laboratory, CUHKSZ-Boyalife Regenerative Medicine Engineering Joint Laboratory, School of Medicine, The Chinese University of Hong Kong, Shenzhen 518172, China

**Keywords:** RPA–CRISPR integrated assay, colorimetric biosensor, microfluidic paper-based analytical device, HPV16 E7 detection, point-of-care diagnostics

## Abstract

Early detection of high-risk human papillomavirus (HPV), particularly HPV16 E7, is critical for cervical cancer prevention. Here, we report a novel, portable, and instrument-free biosensing platform that integrates recombinase polymerase amplification (RPA) with CRISPR/Cas12a-mediated detection on a microfluidic paper-based analytical device (μPAD) for colorimetric, visual readout of double-stranded DNA (dsDNA). The μPAD features seven functional zones, including lyophilized RPA and CRISPR reagents, and immobilized streptavidin and anti-FAM antibodies for signal generation. Upon target recognition, Cas12a’s trans-cleavage activity releases biotinylated-FAM-labeled reporters that form a sandwich complex with gold nanoparticle (AuNP)-conjugated anti-FAM antibodies, producing a visible red signal at the test zone. The gray value of the colorimetric signal correlates linearly with target concentration, enabling the quantitative detection of HPV16 E7 dsDNA down to 100 pM within 60 min. The assay demonstrated high accuracy and reproducibility in spiked samples. By combining isothermal amplification, CRISPR specificity, and paper-based microfluidics, this platform offers a rapid, low-cost, and user-friendly solution for point-of-care HPV screening in resource-limited settings. This work advances the integration of CRISPR diagnostics with μPAD, paving the way for scalable point-of-care molecular diagnostics beyond HPV.

## 1. Introduction

HPV is a group of more than 200 related viruses, with at least 14 types classified as high-risk for causing cancer. High-risk HPV types, particularly HPV16 and HPV18, are responsible for approximately 70% of cervical cancer cases [1,2,3]. Persistent infection with these high-risk HPV types is the primary risk factor for the development of cervical cancer, as it can lead to precancerous lesions that may progress to invasive cancer if not detected and treated early [4]. The relationship between HPV and cervical cancer underscores the critical importance of early detection and screening [5]. Regular screening for HPV is essential because many infections are asymptomatic and can progress without noticeable symptoms until the advanced stages of the disease [6,7]. While current screening methods, such as pap smears and PCR-based HPV DNA testing are highly effective in clinical settings, their reliance on centralized laboratories, trained personnel, and expensive instrumentation limits accessibility in low-resource and rural regions [8,9]. There is an urgent need for point-of-care (POC) diagnostic platforms that are rapid, low-cost, instrument-free, and capable of delivering quantitative, molecular-level results without cold-chain logistics or specialized training.

Recent advances in CRISPR-based nucleic acid detection have revolutionized POC diagnostics by offering high specificity, sensitivity, and isothermal operation, enabling detection at room temperature within minutes [10]. The CRISPR/Cas12a system, in particular, exhibits collateral cleavage activity upon target recognition, allowing signal amplification and versatile readout formats, including fluorescence, electrochemical, surface enhanced Raman spectra, and colorimetric detection [11,12,13]. Moreover, CRISPR-based assays often deliver results within an hour, enabling timely decision-making in outbreak scenarios [14]. A promising development in this field is the integration of CRISPR with isothermal amplification techniques like RPA [15]. This combination enhances sensitivity by amplifying low-abundance nucleic acids and eliminates the need for thermal cycling, simplifying workflows and making diagnostics more accessible in resource-limited settings [16,17]. Additionally, the rapid amplification of RPA paired with CRISPR’s quick detection enables short assay times, while its potential for multiplexing allows for the simultaneous detection of multiple pathogens, addressing co-infections and complex diagnostic needs [7]. Together, these technologies offer a powerful, streamlined approach to pathogen detection, with applications ranging from POC diagnostics to outbreak response.

Microfluidic paper-based analytical devices (μPADs) have emerged as a powerful platform for POC testing, offering portability, low cost, and ease of use [18,19]. Their capillary-driven fluidics and compatibility with lyophilized reagents enable “sample-to-answer” workflows suitable for disease early diagnosis [20], making them ideal for POC, especially in resource-limited settings [21]. However, most μPAD-based nucleic acid assays still rely on the well-established lateral flow test strips or lack quantitative output, limiting their analytical rigor and clinical utility. The nucleic acid signal amplification normally happens in the tube, followed by the signal display on the test strip. Thus, they normally need a complicated reagent process procedure, and have limited sensitivity. Integrating CRISPR/Cas12a with μPADs presents a compelling opportunity to combine molecular specificity and sensitivity with paper-based simplicity.

Here, we report the development of a RPA–CRISPR/Cas12a biosensor embedded in a multi-zone μPAD for direct, quantitative, and visual detection of HPV16 E7 dsDNA. Our platform features lyophilized reagents, on-chip purification, and a colorimetric signal generated via AuNP-antibody sandwich formation, enabling quantification by grayscale analysis or smartphone imaging [22]. We systematically optimized surface chemistry, reagent concentrations, and fluidic control to achieve a limit of detection of 100 pM within 60 min, with excellent accuracy and reproducibility. This work bridges the gap between high-fidelity molecular diagnostics and scalable paper microfluidics, offering a robust, user-friendly, and instrument-free solution for HPV screening that can be readily adapted for other infectious disease or environmental monitoring applications. Previously, we developed an integrated quantitative florescence RPA–CRSIPR/Cas12a biosensor on μPADs for the detection of HPV16 E7 dsDNA [23]. However, few studies have successfully implemented a fully integrated quantitative colorimetric CRSIPR assay on a single with on-device sample processing.

Early RPA–CRISPR implementations used fluorescent readouts, which required a fluorescence reader, optical alignment, and laboratory infrastructure. In this study, we developed a fully colorimetric μPAD technology, where the analytical signal can be directly interpreted by the naked eye while experimental results are quantified using a color analyzer [24]. Our platform uniquely integrates on-paper RPA amplification, paper-based CRISPR/Cas12a activation, and AuNP-based colorimetric detection into a single device, enabling a completely instrument-free workflow and portable test strips that do not require a fluorescence reader.

## 2. Experimental Section

### 2.1. Materials and Chemicals

The 6× loading buffer (D2041) was purchased from US Everbright Inc. (Juno Beach, FL, USA). DNA ladders (3592A), Exonuclease I (2650A), Exonuclease III (2170A), Taq DNA polymerase (R001A), and T4 ligase (2011B) were purchased from Takara (Kyoto, Japan). SYBR Green I (SY1020-50) and D-trehalose anhydrous (IT0870) were purchased from Solarbio (Beijing, China). Whatman filter paper No.1 (WHA1001150), and Whatman filter paper No.4 (WHA1004125) were purchased from Merk (Shanghai, China). Absorption pads (Kinbio, Shanghai, China, CH37) and backing pads (Kinbio, China, SM31-25) were used to make μPADs. LbCas12a (M0653T) was purchased from New England Biolabs (Ipswich, MA, USA). Magnesium chloride (M2670-100 g), glycine (G8898-500 g), Triton X-100 (93443-100 mL), Tween20 (P9416-50 mL), bovine serum albumin (B2063-50 g), polyethylene glycol (PEG, 8210371000), poly-L-lysine (P5899-5 mg), HAuCl_4_·3H_2_O (1015820001), and streptavidin (S4762-1 mg) were purchased from Sigma. EDTA (E196386-100 mL) and sucrose (S112231-500 g) were purchased from Aladdin (Shanghai, China). The pair of anti-FAM antibodies (3D7, 9D7) were purchased from Biocare (Zhuhai, China). Dithiothreitol was purchased from Thermo Fisher Scientific (Waltham, MA, USA). TwistAmp Basic kit RPA (TABAS03KIT) was purchased from TwistDx Limited (Cambridge, UK). Streptavidin magnetic beads (22308-1) were purchased from Beaver (Suzhou, China). All synthesized oligonucleotides were synthesized by Sangon Biotech (Shanghai, China), as shown in Table S1. The sequence of HPV16 E7 cDNA was from GenBank (GenBank: AF477385.1).

### 2.2. Apparatus

DNA concentration was measured by Thermo Scientific^TM^ NanoDrop^TM^ One Microvolume UV–Vis Spectrophotometer. Fluorescence was recorded by a microplate reader (PerkinElmer EnVision multimode plate reader #2105-0010). Gel electrophoresis was performed using a Bio-Rad Electrophoresis system (Herucles, CA, USA) and imaged using Jiapeng JP-BL100 blue-light transilluminator (Shanghai, China) or a TIANGEN TGel Image System (Beijing, China). The colorimetric intensity on the µPADs was detected using a home-made colorimetric detector. RT-qPCR was performed using a BioRad CFX96 qPCR system (Laboratory, CA, USA).

### 2.3. Design and Fabrication of the Colorimetric μPADs

**Figure 1:**
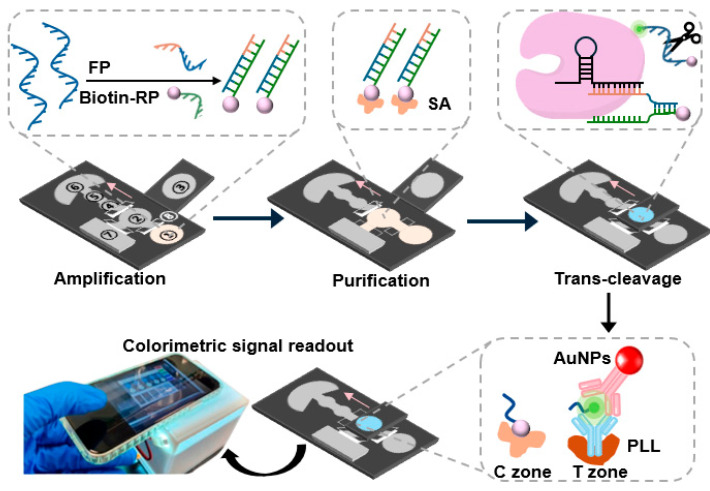
Workflow of the μPADs with colorimetric signal output. The structure of the μPADs: ① RPA zone: primers and other RPA reagents, ② Purification and ④ C zone: PLL, streptavidin, ③ CRISPR zone: CRISPR/Cas reagents, ⑤ T zone: PLL, anti-FAM Ab, ⑥ and ⑦ Waste zone, and ⑧ Valve.

The μPADs were fabricated using Whatman filter paper. μPADs were prepared by using a wax printer (Xerox ColorQube 8570) based on the structure in Figure 1. The key reagents and function of different regions in μPADs is detailed in Table S2. Region 1 is used for sample addition and RPA reaction. Region 2 is designated for purification, where the amplified dsDNA is immobilized, and unreacted primers are washed away. The absorption pad to its left absorbs waste liquid from the purification process. Region 3 is pre-loaded with CRISPR-associated reagents. Regions 4 and 5 are pre-coated with streptavidin and anti-FAM antibodies. Streptavidin binds to biotin, while the anti-FAM antibody interacts with AuNP-Ab and FAM to form a sandwich structure. Region 6 displays the colorimetric signal, and Region 7 drives flow and absorb excess liquid. The gray intensity of these regions reflects the concentration of the target sample. The fan-shaped area at the top serves as a waste liquid absorption zone. The black areas in the diagram are wax printed. The wax permeates the filter paper upon melting, creating hydrophobic boundaries to ensure liquid flows within the white areas. After printing, the filter paper is heated at 120 °C for 2 min to allow wax penetration. The filter paper is then adhered to a backing and stored in a dry environment. Because the reaction area is confined by a wax-printed hydrophobic barrier, which prevents lateral spreading and helps maintain a small reaction volume, evaporation has little effect on the RPA or Cas12a reactions in our μPAD. In addition, the μPAD remains covered in a humid environment, and the reaction times are relatively short (20 min for RPA and 30 min for Cas12a), which further reduces evaporation loss.

### 2.4. PCR Amplification and Agarose Gel Electrophoresis

To confirm that the designed primers were capable of amplifying the target DNA sequence, a polymerase chain reaction (PCR) system was prepared with carefully optimized components. A total of 99.5 μL of template-free reaction mixture was first assembled, consisting of 10 μL of 10× Taq buffer, 8 μL of 2.5 mM dNTP solution, 6 μL of 25 mM MgCl_2_, 0.5 μL of Taq polymerase (5 U μL^−1^), 10 μL each of the 10 μM forward primer (TD-FP) and reverse primer (Biotin-RP), and 55 μL of RNase-free water. For the experimental group, 0.5 μL of 60 ng μL^−1^ HPV16 E7 gene template was added, whereas the negative control contained an equal volume of RNase-free water instead of the DNA template.

PCR amplification was carried out under the following thermal cycling conditions: an initial denaturation at 94 °C for 5 min; 35 cycles of 94 °C for 30 s, 54 °C for 30 s, and 72 °C for 1 min; followed by a final extension step at 72 °C for 5 min. The reaction products were then maintained at 4 °C until analysis. The PCR products were verified by agarose gel electrophoresis (1%). For this procedure, 10 μL of each PCR product was combined with 2 μL of 6× loading buffer and 1 μL of 10× SYBR Gold dye. A total of 10 μL of this mixture was loaded into each gel well and electrophoresed in 1× TAE buffer at 100 V for approximately 30 min. After electrophoresis, the gel was rinsed with distilled water and visualized under a UV imaging system. The desired E7 double-stranded DNA band was excised and purified using a commercial gel extraction kit following the manufacturer’s protocol. The purified fragment (193 bp) was quantified using a Nanodrop spectrophotometer, diluted in RNase-free water, and stored at −20 °C for future experiments. In this study, a larger PCR reaction volume (100 μL) was used to obtain a sufficient amount of HPV16 E7 amplification product in a single amplification. This PCR product was not only used for gel electrophoresis verification but also as a standard DNA template for subsequent RPA amplification and CRISPR/Cas12a quantitative calibration experiments, thereby avoiding repeated amplification and reducing batch-to-batch variation. Despite the larger reaction volume, a gel recovery step is still required to ensure that the standard DNA has high purity and a clear fragment length. This purification process can effectively remove residual primers, non-specific amplification products, and enzyme components, preventing them from interfering with the subsequent RPA amplification efficiency and Cas12a-mediated cleavage reactions, thereby ensuring the reliability and reproducibility of subsequent experimental results.

### 2.5. RPA–CRISPR/Cas12a-Based Detection of HPV16 E7 dsDNA in Solution

To validate the ability of the designed primers to amplify the HPV16 E7 target sequence, a recombinase polymerase amplification (RPA) assay was established. (i) The RPA master mix is assembled and initiated by adding MgOAc, (ii) the amplification is terminated by heating, (iii) the products are briefly purified using streptavidin-coated magnetic beads to remove primers and potential inhibitors, and (iv) the purified amplicons are then transferred into the Cas12a reaction mixture for fluorescence detection. Each of these steps is performed manually because the solution-phase assay serves as a bench-top validation of the biochemical components, prior to their integration onto the μPAD, where these operations are automated by the device design. A 46.5 μL template-free RPA master mix was first assembled, consisting of 29.5 μL rehydration buffer, 2.4 μL of 10 μM forward primer (TD-FP), 2.4 μL of 10 μM reverse primer (Biotin-RP), and 12.2 μL of RNase-free water. Subsequently, 1 μL of HPV16 E7 double-stranded DNA (dsDNA) template was added to initiate amplification, while an equal volume of RNase-free water served as the negative control. The reactions were initiated by adding 2.5 μL of 280 mM magnesium acetate (MgOAc), gently mixed, and incubated at 39 °C for 20 min. The amplification was terminated by heating at 95 °C for 10 min prior to purification. To eliminate residual primers and potential inhibitors that could interfere with the CRISPR/Cas12a assay, the RPA products were purified using streptavidin-coated magnetic beads (MBs). A total of 3 μL of MBs (10 mg mL^−1^) were prewashed twice with 200 μL of B&W buffer (5 mM Tris-HCl, 0.5 mM EDTA, 1 M NaCl, 0.5% Tween-20, pH 7.4) to remove surfactants. After discarding the supernatant, 5 μL of the RPA reaction mixture was added to the beads and incubated at room temperature for 15 min under gentle rotation to allow binding. The beads were magnetically separated, the supernatant discarded, and the pellet was washed twice with 200 μL of B&W buffer to ensure clean isolation of amplicons. The CRISPR/Cas12a detection assay was then performed in a total volume of 25 μL. The reaction mixture contained 15.6 μL of nuclease-free water, 2.5 μL of 10× HOLMES buffer (20 mM spermine, 400 mM Tris-HCl, 60 mM MgCl_2_, 10 mM DTT, 400 mM glycine, 0.01% Triton X-100, and 4% PEG20000, pH 8.5), 0.625 μL of 1 μM LbCas12a nuclease, 1.25 μL of 1 μM crRNA, and 2.5 μL of 1 μM single-stranded DNA (ssDNA) fluorophore-quencher reporter. Following removal of the wash buffer, the bead-bound RPA product was resuspended in the CRISPR/Cas12a reaction mixture. The reactions were incubated at 37 °C for 20 min, after which fluorescence signals (Ex = 570 nm, Em = 615 nm) were recorded using a multifunctional microplate reader. All assays were conducted in duplicate, and data are presented as the mean ± standard deviation (SD).

### 2.6. Preparation of the AuNP Probes

A monodisperse suspension of AuNPs (40 nm) was made using the bottom-up approach. Before usage, the produced AuNPs were kept at 4 °C. Before dispersing in Milli-Q water, AuNPs were taken out of the refrigerator. The AuNP solution was made to have a pH of 8.4 by adding 0.25 M of potassium carbonate (16 μL). After adding the anti-FAM antibody (9B7, 20 μg) to the AuNP solution (1 mL), the mixture was incubated for 30 min in a rotating incubator. To inhibit the unbinding sites on AuNPs, a 1% BSA solution was added after incubation, and the mixture was then incubated for a further 25 min. For 15 min, the mixture was centrifuged at 4 °C using 8000 rcf. After discarding the supernatant, the AuNP probes were reconstituted using the resuspension buffer (2 g of sucrose, 2 g of D-Trehalose anhydrous, 10 mg of PVP, 10 mg of BSA, 50 μL of Tween20, and 10 mL of 0.01 M PBS). Before use, the AuNP probes tagged with antibodies were kept at 4 °C.

### 2.7. Detection of the Colorimetric μPADs

In Regions 4 and 5, 0.3 µL of 0.025% PLL was added, and in Region 1, 2 µL of 0.0025% PLL was added to allow streptavidin and anti-FAM Ab to absorb into these regions. The μPADs were then placed at 37 °C for 10 min to allow the moisture to evaporate. Afterward, 3 µL of streptavidin (1 mg mL^−1^) was added to Region 2, 0.3 µL of streptavidin (2 mg mL^−1^) was added to Region 4, and 0.3 µL of anti-FAM Ab (3D7, 1.5 mg mL^−1^) was added to Region 5. These were then dried at 37 °C for 10 min. The streptavidin in Region 2 bound biotin to fix the RPA amplification products in this region for the subsequent CRISPR reaction. The streptavidin in Region 4 bound biotin on the reporter, ensuring the cleaved reporter moved forward and formed a sandwich structure with the anti-FAM Ab in Region 5 and the Ab conjugated to AuNPs. To reduce nonspecific adsorption and help the liquid flow more smoothly on the μPADs, 15 µL of blocking buffer (2% BSA, 0.25% Tween 20, 2% sucrose, 0.01 M PBS, pH 7.4) was added to the μPADs to block excess binding sites, and they were incubated at 37 °C for 10 min. Next, 2.95 µL of Rehydration buffer, 2.4 µL of TD-FP, and 2.4 µL of RP were added to Region 1 of the pretreated μPADs. Then, 2.5 µL of 10× NEB buffer, 1.25 µL of crRNA, and 0.625 µL of LbCas12a Nuclease were added to Region 3. The μPADs were then frozen at −20 °C for 30 min. After freezing, a freeze-dryer was used to lyophilize the μPADs, which were stored at −20 °C for later use [24].

### 2.8. Detection of HPV16 E7 dsDNA on μPADs

The sample (5 μL) and 0.25 μL 280 mM MgOAc were added to reaction zone 1. RPA amplification was initiated immediately upon the addition of the sample to the sample zone. Then, the valve was opened, allowing the liquid to flow into the purification zone. The RPA amplicons were captured in the purification zone, while any unreacted primers were directed to the waste zone. Subsequently, the lift-up zone containing CRISPR/Cas12a reagents was folded over the purification zone, and all valves were closed to initiate the CRISPR/Cas reaction. This cleavage event led to the release of biotin-ssDNA-FAM. After 30 min, upon completion of the CRISPR/Cas reaction, the CRISPR/Cas zone was lifted away from the purification zone, and gold nanoparticle (AuNP)-labeled anti-FAM antibodies were added to the purification zone while keeping the colorimetric output valve open [25]. The biotin end of the linear reporter generated from the cleavage reaction bound to streptavidin in the C zone, while the FAM label on the opposite end formed a sandwich structure with the pre-coated anti-FAM antibodies in the T zone and the AuNP-conjugated antibodies. After 8 min, a red color developed in both the C and T zones, which was quantified using ImageJ software (1.8.0). Table S1 lists the sequences used in this study. μPADs used a physical paper-based valve. The valve is operated manually by folding or unfolding the corresponding paper layer, which temporarily blocks or permits fluid flow through the wax-defined channels. μPADs contain multiple such foldable valves, with each positioned between functional regions (e.g., between the RPA zone, purification zone, and CRISPR/Cas zone). During the CRISPR reaction step, every foldable valve on the device is placed in the closed position to isolate the reaction zone and prevent unintended flow. The C zone refers to the “control” zone, where streptavidin captures the biotin-labeled reporter after Cas12a cleavage. This zone confirms that the device functioned correctly and provides an internal control for the colorimetric readout.

## 3. Results and Discussion

### 3.1. Performance of the RPA–CRISPR/Cas12a Biosensor in Solution for the Detection of HPV16 E7

**Figure 2:**
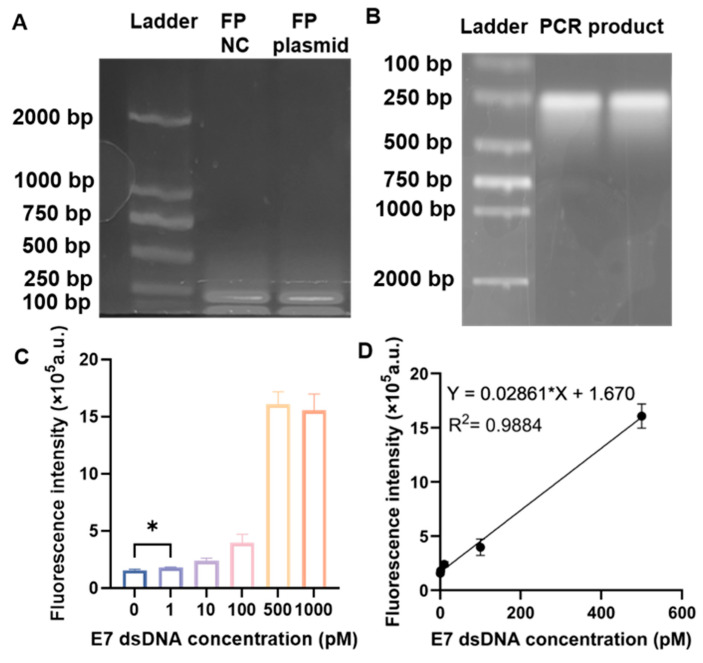
Feasibility of RPA–CRISPR/Cas12a biosensor in a tube-based assay. (**A**) Agarose gel image of RPA products. (**B**) Agarose gel image of PCR products. (**C**) Sensitivity analysis of RPA–CRISPR/Cas12a biosensor for detection of HPV16E7dsDNA in tube (1 pM–1000 pM) (* indicates a statistically significant difference between the indicated groups (*p* < 0.05).) (*n* = 3, error bar represents ± s.d.). (**D**) Calibration curve of RPA–CRISPR/Cas12a biosensor for detection of HPV16E7dsDNA in tube.

To determine the detection sensitivity of E7 dsDNA, fluorescence-based assays were first performed. Forward and reverse RPA primers were designed to amplify the cDNA reverse-transcribed from HPV16 E7 mRNA. As shown in the RPA agarose gel image (Figure 2A), a distinct amplification band was observed, indicating that the RPA reaction proceeded as expected and the target cDNA was successfully amplified in the presence of the template. To further confirm whether the RPA amplicon could effectively trigger the subsequent CRISPR/Cas12a reaction, a plasmid containing the E7 gene was amplified by PCR. The PCR product visualized on an agarose gel exhibited a single band near 200 bp (Figure 2B), consistent with the expected HPV16 E7 fragment size of 195 bp, confirming the successful amplification of the E7 gene. Based on previously optimized conditions, the HOLMES buffer was selected for the fluorescence detection of E7 dsDNA across a concentration range of 0–1000 pM. The fluorescence intensity increased proportionally with the concentration of HPV16 E7 double-stranded DNA (Figure 2C). When the target concentration exceeded the threshold of 1 pM, a significant enhancement in fluorescence was observed, indicating successful activation of the Cas12a-mediated trans-cleavage reaction. These findings demonstrate that the RPA–CRISPR/Cas12a system enabled the highly sensitive and specific detection of HPV16 E7 with a detection limit of approximately 1 pM, while fluorescence intensity plateaued beyond 500 pM, suggesting signal saturation at higher target concentrations. The standard calibration curve was established using a series of gradient concentrations of the target DNA templates. As shown in Figure 2D, the fluorescence intensity increased proportionally with the concentration of the standard samples, indicating a strong linear relationship between signal intensity and DNA quantity. The linear regression analysis revealed an excellent correlation (R^2^ = 0.9884). The lower-concentration samples produced faint but clearly detectable fluorescence, whereas higher concentrations reached a plateau, suggesting that the detection system approached saturation at the upper limit. This calibration curve was subsequently employed to estimate the concentration of unknown samples, which displayed fluorescence intensities corresponding to the higher end of the standard range. The consistency of triplicate measurements confirmed the reproducibility and accuracy of the quantification method. In Figure 2D, 5 concentration points were used to construct the calibration curve (0 pM, 1 pM, 10 pM, 100 pM, and 1000 pM). These concentrations were selected to span the dynamic range of the assay while avoiding saturation at higher levels. The R^2^ value was slightly below 0.99 because it reflects a biological assay with enzymatic amplification, where some variation is expected, especially at the low-concentration end. Despite this, the linear correlation remained strong and sufficient for quantitative interpretation within the tested range, as also supported by the reproducibility of triplicate measurements.

### 3.2. AuNP-Ab Mobility Test on the μPADs

**Figure 3:**
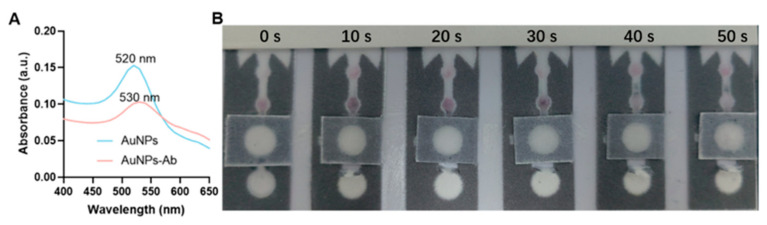
(**A**) UV–Vis spectra of bare AuNPs and anti-FAM antibody-labeled AuNPs. (**B**) Testing the mobility of AuNP-Ab on the μPADs under different valve opening time conditions.

To achieve fast and convenient detection, the μPAD was designed to output colored signals. In the µPAD platform, a physical valve-closing strategy was employed to control fluid flow. As shown in Figure 3A and Table S2, λ_max_ for bare AuNPs was observed at 520 nm, while λ_max_ for the AuNP-Ab conjugates shifted to 530 nm [26]. When antibodies are attached to the nanoparticles, a protein layer forms around the AuNPs, which (i) slightly increases the particle’s hydrodynamic size, (ii) alters the local refractive index, and (iii) reduces the effective surface plasmon resonance efficiency. These effects commonly lead to a modest decrease in absorbance intensity compared with bare AuNPs, even though the red-shift from 520 nm to 530 nm confirms successful conjugation. This shift in the absorption peak indicates that the antibody was successfully conjugated to AuNPs. The change in the peak wavelength is a characteristic signature of nanoparticle surface modification, confirming that the anti-FAM antibody was effectively attached to AuNPs. AuNP-Ab was added to six µPADs prepared in the same batch. To prevent unreacted primers from interfering with the subsequent CRISPR/Cas12a cleavage and causing false positives, the valve closing time should not exceed 1 min, so the valve at the sample position was closed at 0 s, 10 s, 20 s, 30 s, 40 s and 50 s (Figure 3B). It was observed that AuNP-Ab flowed well on all six µPAD platforms, with the best flow at 30 s. Therefore, in subsequent biosensor detection, the valve was closed at 30 s after add sample. This flow data also demonstrated the feasibility of using AuNP-Ab biosensing on µPADs.

### 3.3. Performance of RPA–CRISPR/Cas12a Colorimetric Biosensor on µPADs for HPV16 E7 Detection

**Figure 4:**
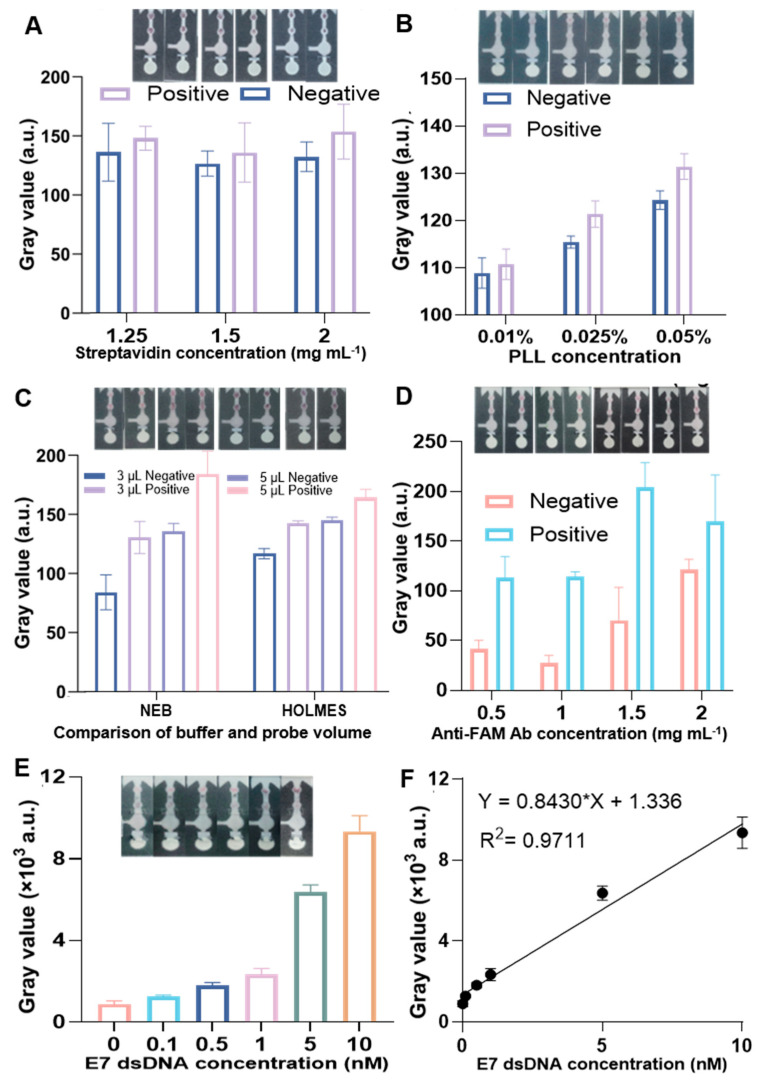
Performance of RPA–CRISPR/Cas12a colorimetric biosensor on µPADs. (**A**) Result of streptavidin concentration optimization. (**B**) Result of PLL concentration optimization. (**C**) Result of the comparison of two buffers and AuNP-Ab volume. (**D**) Result of anti-FAM Ab concentration optimization. (**E**) Sensitivity of the RPA–CRISPR/Cas12a biosensor on µPADs (*n* = 3, error bar represents ± s.d.). (**F**) Calibration curve of the RPA–CRISPR/Cas12a biosensor on µPADs (*n* = 3, error bar represents ± s.d.).

In the application of μPADs, performing three or more replicate experiments is a standard scientific practice to enhance data reliability, validate result significance, and ensure reproducibility [26,27,28]. Based on the verification of the properties of the μPADs, we directly tested whether the RPA–CRISPR/Cas12a biosensor worked on the pad after RPA amplification; the amplicons were immobilized in the CRISPR reaction zone, where they triggered trans-cleavage. This cleavage event resulted in the release of bio-ssDNA-FAM. The biotin-modified strand binds to streptavidin in the C zone, while the FAM label on the other end of the amplicon forms a sandwich structure with an antibody on the T zone and AuNP-conjugated antibodies. As a result, the gray value in the T zone is directly proportional to the concentration of the target DNA. This provides a simple yet effective method for quantifying the presence of the target, with the color intensity offering a clear visual indication of the DNA concentration. Subsequently, we optimized several parameters on the µPADs to improve the assay’s performance. As shown in Figure 4A, we optimized the concentration of streptavidin in the C zone. It was evident that when the concentration of streptavidin was 2 mg mL^−1^, the difference in gray values between the positive and negative results was maximized. Next, we optimized the concentration of PLL, which is positively charged and has a strong affinity for negatively charged proteins. As shown in Figure 4B, the increasing concentration of PLL significantly increased the signal-to-noise ratio. However, the SNR improvement became minimal when the PLL concentration exceeded 0.025%. Thus, we selected 0.025% as the optimal concentration for further experiments. Based on this observation, we selected this concentration for subsequent experiments to ensure the best signal differentiation. Given that the HOLMES buffer contained high-molecular-weight components such as PEG20000, which may interfere with the colorimetric detection of the µPADs [29], we compared the performance of the assay using HOLMES buffer and NEB buffer. We assessed the effects of adding different volumes of AuNP-Ab during the cleavage reaction. As shown in Figure 4C, when NEB buffer was used for the cleavage reaction, the difference in gray values between the positive and negative results was more pronounced. Moreover, when 3 µL of AuNP-Ab was added, the signal difference between the positive and negative results was even larger. This might be due to the increased concentration of AuNP-Ab causing some non-specific adsorption. Therefore, we used NEB buffer as the CRISPR reaction solution for subsequent experiments and added 3 µL of AuNP-Ab. We then optimized the concentration of anti-FAM Ab in the T zone (Figure 4D). We found that when the concentration was 1.5 mg mL^−1^, the signal-to-noise ratio was maximized. Higher concentrations of anti-FAM Ab led to potential non-specific binding, which could negatively affect the results. Therefore, we chose 1.5 mg mL^−1^ as the optimal concentration for the anti-FAM antibody in subsequent experiments. Finally, we evaluated the sensitivity of this system by testing different concentrations of HPV16 E7 dsDNA (100 pM, 500 pM, 1 nM, 5 nM, 10 nM). As expected, the gray value increased with the concentration of the target, demonstrating that the assay’s sensitivity on µPADs was as low as 100 pM (Figure 4E). A calibration curve was established in Figure 4F to quantitatively evaluate the relationship between color signal intensity and E7 dsDNA concentration. The color signal intensity increased steadily with the concentration of E7 dsDNA, showing an approximately linear relationship within the tested range. Regression analysis results indicated a good linear correlation between the two (R^2^ = 0.9711), suggesting that the color signal responds proportionally to the analyte concentration. At low concentrations, the signal was weak but clearly above the background, confirming the high sensitivity of the detection system. As the concentration increased, the signal enhancement trend was significant, while the signal in the highest concentration group tended to saturate, indicating that the detection system reached its quantitative limit at this point. The analytical accuracy of the developed colorimetric RPA–CRISPR/Cas12a assay was assessed through recovery experiments using standard HPV E7 dsDNA solutions at three concentration levels (0.1, 1, and 5 nM). As summarized in Table 1, the gray values exhibited a concentration-dependent increase, confirming the assay’s reliable quantitative response within the tested range. The calculated recoveries were 101.0%, 96.2%, and 97.2% for the respective concentrations, yielding an average recovery of approximately 98.1%. All recovery values fell within the generally acceptable range of 95–105%, indicating good analytical accuracy with minimal systematic deviation. In our case, recoveries >100% likely arise from matrix-induced signal enhancement or slight overestimation during calibration, both of which can increase the measured signal relative to the nominal spiked concentration. Moreover, the low variability among the three levels suggests excellent reproducibility under the current reaction conditions. These results demonstrate that the proposed method possesses high accuracy, stability, and quantitative reliability for detecting HPV E7 dsDNA, and it provides a solid basis for further validation in complex biological or clinical matrices. We also compared the proposed method with other reported CRISPR/Cas biosensors on different platforms (Table S3). It shows herein that CRISPR/Cas on μPADs is cost-effective without the need of a large instrument, demonstrating massive potential in point-of-care testing.

### 3.4. Limitations and Outlook

Despite the advantages of the proposed μPAD-based RPA–CRISPR/Cas12a colorimetric platform, several limitations should be acknowledged. First, compared with fluorescence-based CRISPR assays, the colorimetric readout inherently exhibits a narrower dynamic range and relatively lower sensitivity, which may limit its applicability in scenarios requiring ultra-trace detection. Second, although grayscale analysis enables quantitative interpretation, the colorimetric signal can still be influenced by external factors such as ambient lighting conditions, paper substrate variability, and user-dependent operational differences, potentially affecting quantitative accuracy and inter-device reproducibility. Third, the current device relies on manually operated paper-based valves, which, while simple and low-cost, introduce an additional layer of user intervention that may impact consistency in non-laboratory or large-scale deployment settings. Furthermore, although on-chip purification improves specificity by removing unreacted primers, it also adds structural and operational complexity to the device design. Future work will therefore focus on addressing these limitations by improving signal amplification strategies to enhance sensitivity, optimizing paper microfluidic architectures for more robust and automated fluid control, and integrating smartphone-based image acquisition with algorithm-assisted analysis to minimize environmental and user-related variability. Additionally, extending validation to complex clinical samples and exploring fully enclosed, self-actuated device formats will be critical steps toward translating this platform into practical point-of-care diagnostic applications.

## 4. Conclusions

In this work, we present a novel, integrated biosensing platform that combines recombinase polymerase amplification with CRISPR/Cas12a-mediated detection on a microfluidic paper-based analytical device for the rapid, instrument-free, and quantitative detection of HPV16 E7 dsDNA, a critical biomarker for cervical cancer risk. The platform leverages lyophilized reagents, on-chip sample purification, and a colorimetric readout based on AuNP-antibody sandwich formation, enabling visual or smartphone-based quantification with a limit of detection of 100 pM and a linear dynamic range up to 10 nM (R^2^ = 0.9711). Optimized surface chemistry, including PLL-assisted immobilization of streptavidin and anti-FAM antibodies, ensures high specificity and signal-to-noise performance. Recovery experiments in spiked samples demonstrated excellent accuracy (average 98.1%) and reproducibility, validating its potential for real-world clinical or field use. By eliminating the need for thermal cycling, external power, or laboratory infrastructure, this μPAD-based assay represents a significant advancement in point-of-care molecular diagnostics. It provides a scalable, low-cost, and user-friendly template for detecting nucleic acid biomarkers in resource-limited settings, bridging cutting-edge CRISPR diagnostics with accessible paper microfluidics for global health applications. Herein, the μPAD-based RPA–CRISPR/Cas12a platform offers simplicity and instrument-free visualization, although its overall sensitivity is lower than that of fluorescence-based CRISPR assays. Future work will focus on automating fluidic control via origami-inspired valve systems and integrating machine learning-powered smartphone apps for real-time, operator-independent signal analysis, advancing toward fully autonomous, field-deployable diagnostic platforms.

## Figures and Tables

**Table 1:** Result of HPV E7 dsDNA recovery.

Amount Added (nM)	Gray Value (×10^3^ a.u.)	Amount Found	Recovery (%)
0.1	1.27	0.101	101
1	2.83	0.962	96.2
5	7.16	4.86	97.2

## Data Availability

The original contributions presented in this study are included in the article/Supplementary Materials. Further inquiries can be directed to the corresponding author.

## Supplementary Materials

The following supporting information can be downloaded at: https://www.mdpi.com/article/10.3390/bios16010032/s1, Table S1: The sequences used in this study. Table S2: Key reagents and function of different regions in μPADs. Table S3: Comparison of the proposed method with different CRISPR methods. Figure S1: High-resolution enlarged Figure 4A actual detection images. Figure S2: High-resolution enlarged Figure 4B actual detection images. Figure S3: High-resolution enlarged Figure 4C actual detection images. Figure S4: High-resolution enlarged Figure 4D actual detection images. Figure S5: High-resolution enlarged Figure 4E actual detection images.
