# Supplementary material for: Integrated Colorimetric CRISPR/Cas12a Detection of Double-Stranded DNA on Microfluidic Paper-Based Analytical Devices
Source: Biosensors (Basel). 2026 Jan 1;16(1):32. doi: 10.3390/bios16010032 (PMC12839233; doi:10.3390/bios16010032)
Supplement: Supplementary file 1 [file biosensors-16-00032-s001.zip › biosensors-4012101-supplementary.pdf]

Supplementary Information

CRISPR/Cas12a on Microfluidic Paper-based Analytical Devices for Colorimetric Detection of Double Strand DNA

Zhiheng Zhang, Qiyu Fu, Tiantai Wen, Youmin Zheng, Yincong Ma, Shixian Liu, Guozhen Liu*

Integrated Devices and Intelligent Diagnosis (ID^2) Laboratory, CUHKSZ-Boyalife Regenerative Medicine Engineering Joint Laboratory, School of Medicine, The Chinese University of Hong Kong, Shenzhen, 518172, China

*Corresponding author email: liuguozhen@cuhk.edu.cn

Table S1 The sequences used in this study.

Name	Sequence (5' to3')	Modification
**HPV-16 E7 cDNA**	ATTAACAGGTCTTCCAAAGTACAAATGTCTACGTGTGTGCTTTGTACGCACAACCGAAGCGTAGAGTCACACTTGCAACAAAAGGTTACAATATTGTAATGGGCTCTGTCCGGTTCTGCTTGTCCAGCTGGACCATCTATTTCATCCTCCTCCTCTG	
**TD-FP**	ACACAACCACCCAACACAACCAACCCCATTAACAGGTCTTCCAAA	
**Biotin-RP**	TTTTTTTTTCAGAGGAGGAGGATGAAATAG	5' Biotin
**crRNA**	UAAUUUCUACUAAGUGUAGAUGGGGUUGGUUGUGUUGGGUGG	
**Bio-Reporter**	TTTATTATTT	5' Biotin, 3' 6-FAM
**Reporter**	TTATT	5' Texas Red, 3' BHQ2

Table S2 Key reagents and function of different regions in μPADs.

Regions	Function	Key Reagents	Purpose
**Region 1**	RPA amplification	Freeze-dried RPA mix, PLL	Enables on-paper isothermal DNA amplification.
**Region 2**	Streptavidin capture	Streptavidin	Immobilizes biotin-labeled RPA products for CRISPR cleavage.
**Region 3**	Flow channel	—	Guides amplified DNA to the next zone when valve opens.
**Region 4**	Reporter capture	Streptavidin	Binds biotin-FAM reporters; only cleaved fragments pass through.
**Region 5**	Anti-FAM binding	Anti-FAM antibody	Captures cleaved reporters for colorimetric recognition.
**Region 6**	Colorimetric readout	AuNP-Ab (added during assay)	Generates visual signal upon sandwich formation.
**Region 7**	Waste / absorbent	—	Drives flow and absorbs excess liquid.

Table S3 Comparison of the proposed method with different CRISPR methods.

Method	Detection Mode	Instrumentation Required	Estimated Cost per Test (USD)	Notes	Ref
**This work (µPAD RPA–Cas12a)**	Colorimetric (AuNP)	None	**0.5–1.0**	Low enzyme consumption; fully paper-based	This research
Fluorescence CRISPR/Cas12a assays	Fluorescence	Microplate reader / fluorescence device	2–5	Higher Cas12a and reporter usage; instrumentation cost	1
CRISPR lateral flow strips	LFA colorimetric	None	1.5–3	Require commercial LFA strips and gold antibody reagents	2
Standard RPA–CRISPR bench-top assays	Fluorescence or gel	Fluorescence reader / gel system	3–6	Additional purification or handling steps	3

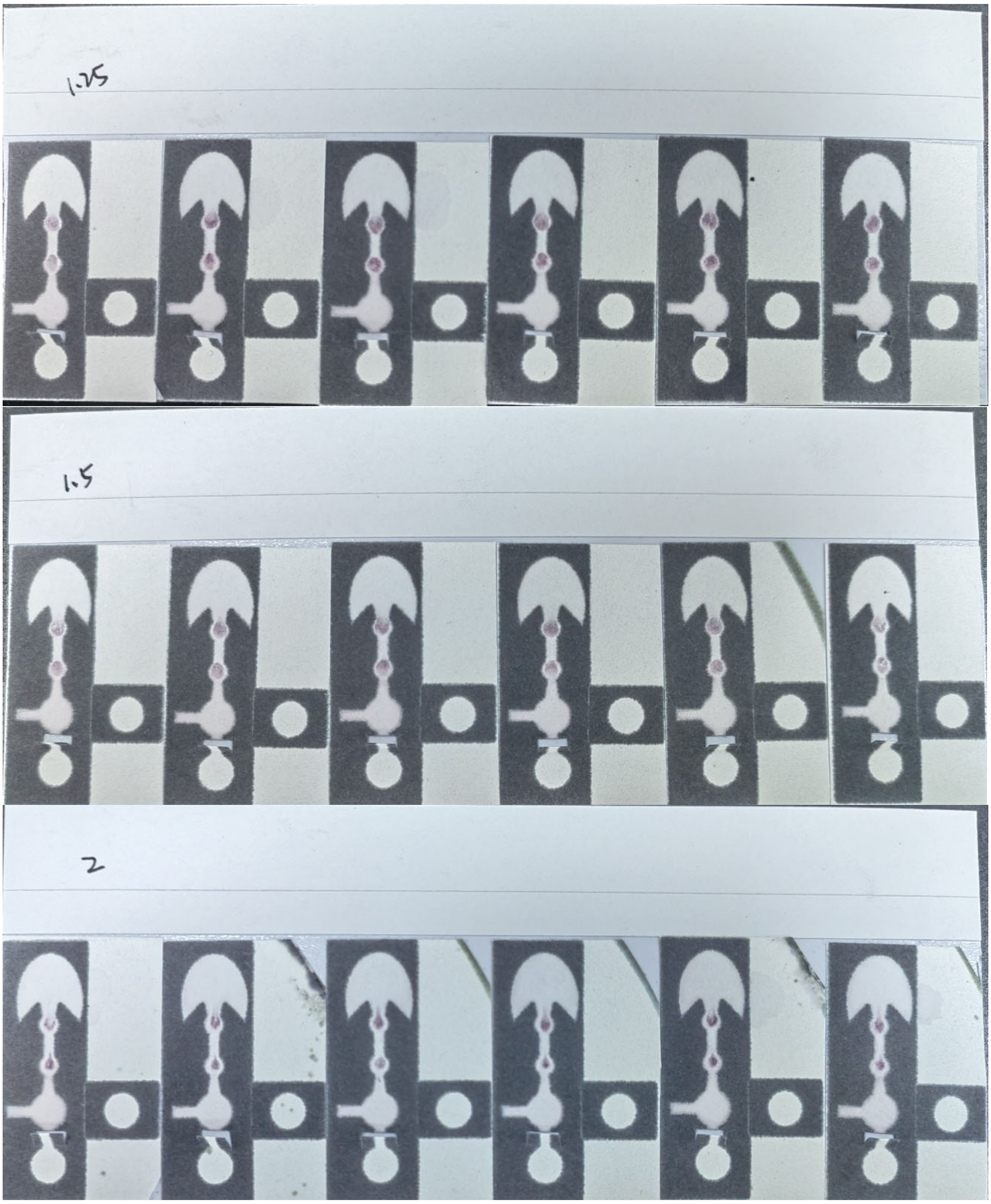

Figure S1. High-resolution enlarged Figure 4A actual detection images.

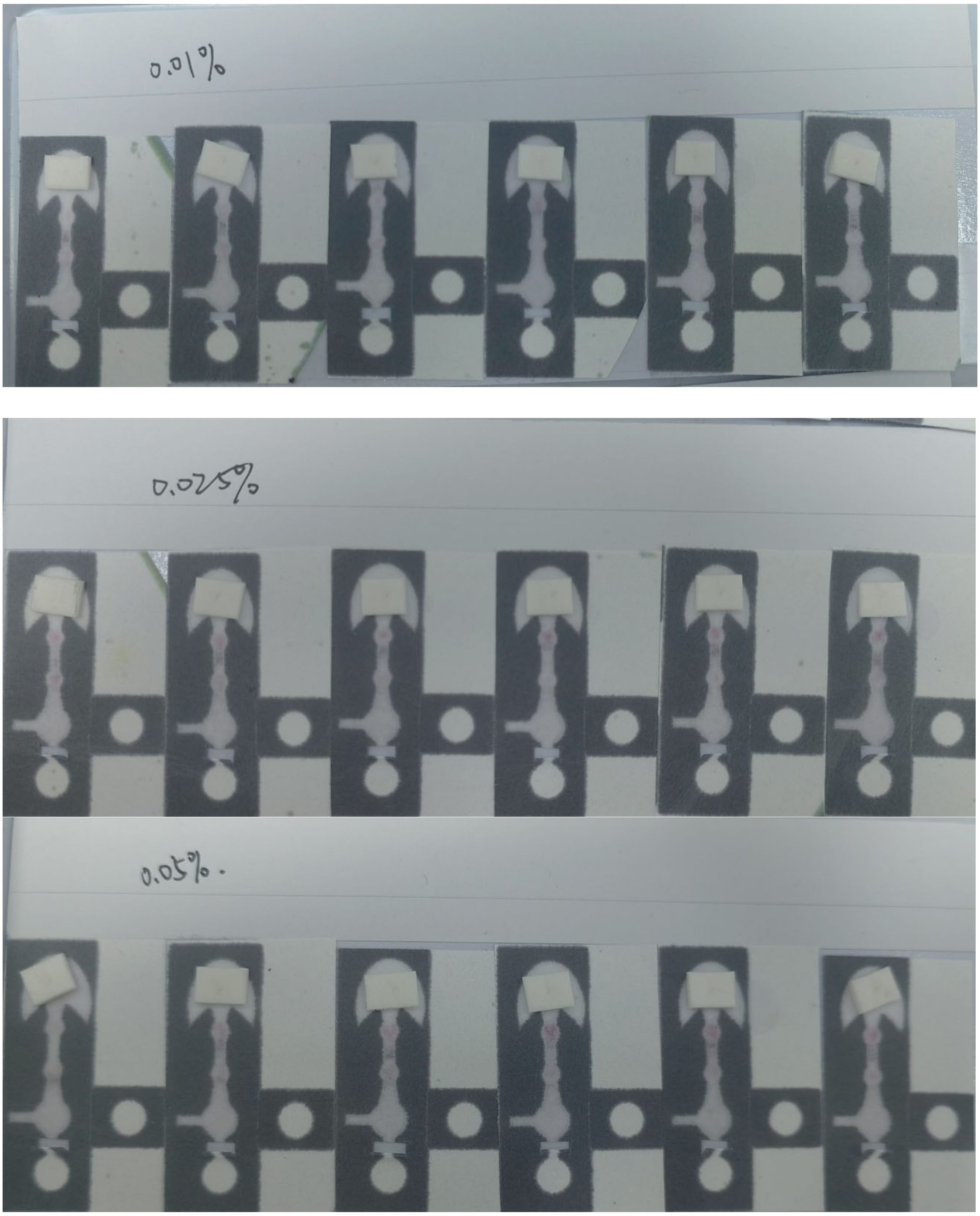

Figure S2. High-resolution enlarged Figure 4B actual detection images.

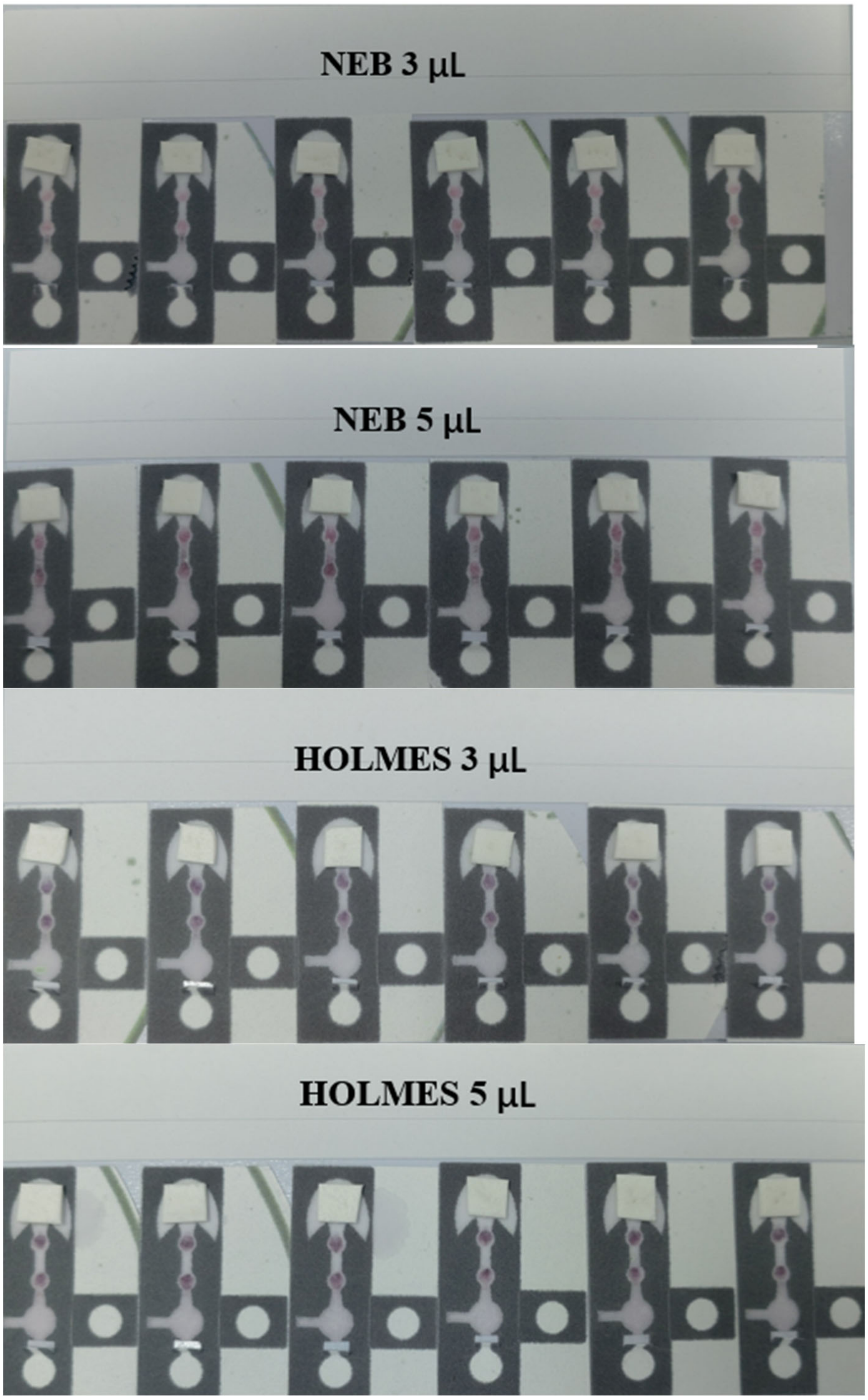

Figure S3. High-resolution enlarged Figure 4C actual detection images.

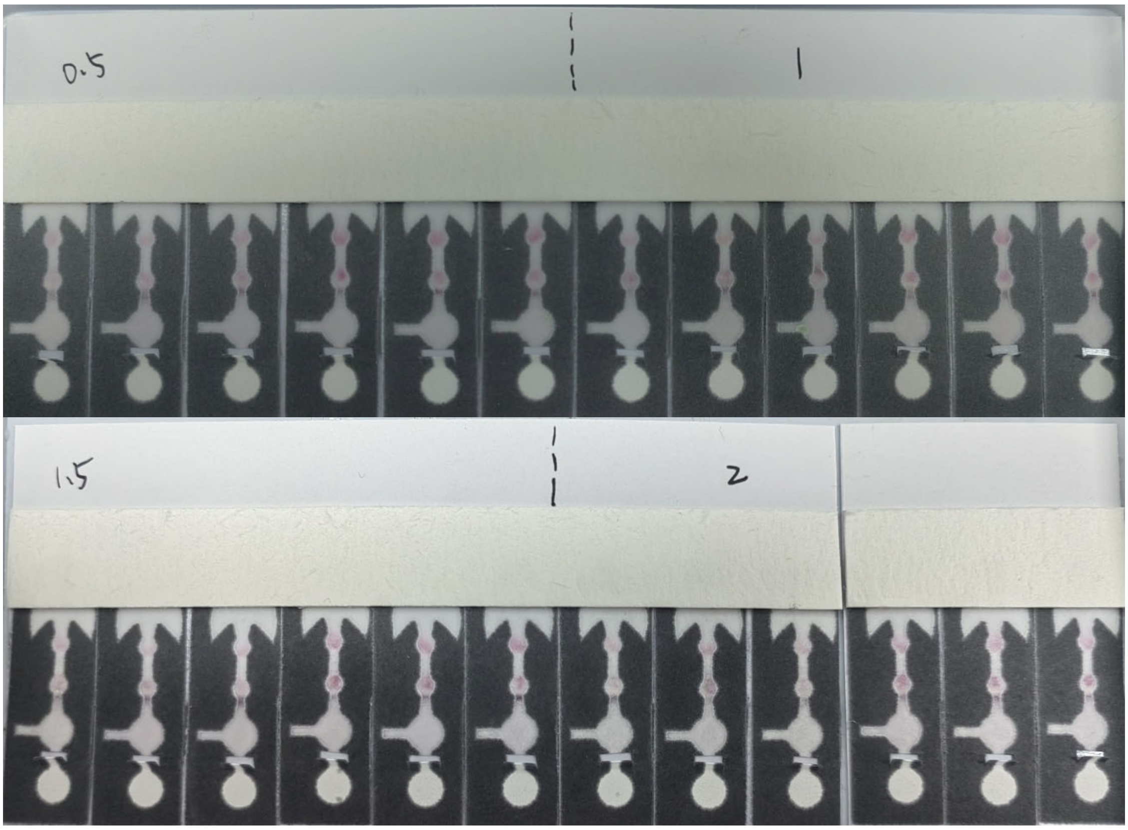

Figure S4. High-resolution enlarged Figure 4D actual detection images.

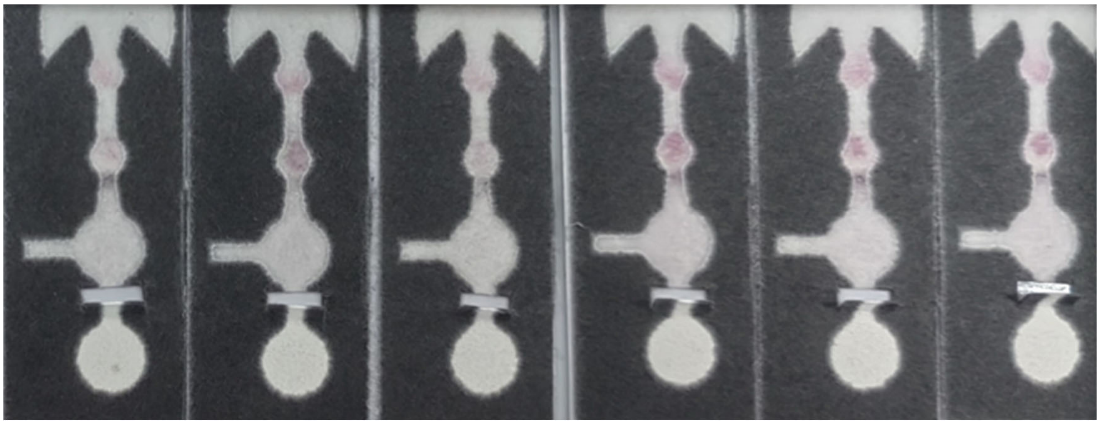

Figure S5. High-resolution enlarged Figure 4E actual detection images.
